# Usefulness of Retinol‐Binding Protein in Predicting Mortality in Patients With Chronic Liver Disease

**DOI:** 10.1002/jgh3.70087

**Published:** 2025-02-08

**Authors:** Yuki Utakata, Takao Miwa, Tatsunori Hanai, Masashi Aiba, Shinji Unome, Kenji Imai, Yohei Shirakami, Koji Takai, Masahito Shimizu

**Affiliations:** ^1^ Department of Gastroenterology/Internal Medicine Gifu University Hospital Gifu Japan; ^2^ Department of Gastroenterology Chuno Kosei Hospital Seki Japan; ^3^ Center for Nutrition Support and Infection Control Gifu University Hospital Gifu Japan; ^4^ Division for Regional Cancer Control Graduate School of Medicine, Gifu University Gifu Japan

**Keywords:** liver cirrhosis, malnutrition, protein synthesis, rapid turnover protein, survival

## Abstract

**Background and Aim:**

Rapid turnover proteins (RTPs), including retinol‐binding protein (RBP), prealbumin, and transferrin, are useful in evaluating dynamic nutritional status. This study aimed to investigate the relationship between serum RTP levels and mortality in patients with chronic liver disease (CLD).

**Methods:**

We evaluated 341 patients with CLD admitted between October 2011 and December 2021. Those with RBP levels below 2.7 mg/dL for males and 1.9 mg/dL for females were included in the low RBP group. Factors associated with mortality and low RBP were evaluated using the Cox proportional hazard regression and logistic regression models.

**Results:**

The median age of the included patients was 67 years, and 48% were male. The median model for end‐stage liver disease (MELD) score was 8 points, and the median RBP, prealbumin, and transferrin levels were 1.5 mg/dL, 11 mg/dL, and 227 mg/dL, respectively. During a median observational period, 23% of the patients died. Multivariate analysis showed that the RBP level (hazard ratio, 0.62; 95% confidence interval [CI], 0.46–0.81) was independently associated with mortality, while prealbumin and transferrin were not. Additional analysis revealed that male sex (odds ratio, 8.62; 95% CI, 2.56–29.00) and albumin level (odds ratio, 0.10; 95% CI, 0.04–0.26) were significantly associated with the low RBP levels in patients with CLD.

**Conclusions:**

The serum RBP level is a dynamic biomarker associated with mortality in patients with CLD, independent of liver functional reserve, and it may be a useful indicator for nutritional intervention in these patients.

## 1 Introduction

Malnutrition is the most common problem in patients with chronic liver disease (CLD), affecting 20%–50% of these patients [1, 2, 3]. In patients with CLD, malnutrition can worsen the functional reserve of the liver, increase mortality rates, and lead to complications such as infection, ascites, and hepatic encephalopathy [2, 4]. Therefore, nutritional assessment is an essential step in identifying patients with CLD who require nutritional intervention to improve their outcomes. Identifying patients with CLD with poor prognosis through appropriate nutritional assessment and the provision of nutritional intervention may improve mortality and their quality of life [4].

According to the guidelines, nutritional interventions for patients with cirrhosis are recommended for those with low albumin levels, worse Child–Pugh class, or sarcopenia [2, 3]. However, these criteria represent relatively gradual markers of nutritional deterioration, and the utility of short‐term nutritional indicators remains to be explored. For instance, although serum albumin levels are commonly used as a biomarker of nutritional status, the half‐life of albumin is approximately 17 days [5]. Therefore, there is an urgent need to identify biomarkers that reflect short‐term deterioration in nutritional status and to investigate their association with prognosis in patients with CLD.

Rapid turnover proteins (RTPs), including retinol‐binding protein (RBP), prealbumin, and transferrin, are synthesized in the liver and have short half‐lives, such as 12 h for RBP, 48 h for prealbumin, and 9 days for transferrin [6]. Due to their short half‐lives, RTPs are commonly used as biomarkers to evaluate protein synthesis and dynamic nutritional status in various conditions [7, 8]. RTPs may be dynamic biomarkers to identify patients at risk of worse outcomes because impaired protein synthesis and worse nutritional status adversely affect the clinical course of patients with CLD [9, 10]. In particular, RBP helps transfer of retinol (vitamin A), which is essential for growth, immunity, sexual reproduction, and vision and is associated with hepatic fibrogenesis and hepatocarcinogenesis [11, 12]. However, the usefulness of RTPs in predicting the prognosis of patients with CLD remains unclear. Therefore, this study aimed to investigate the association between RTPs and mortality in patients with CLD.

## 2 Methods

### 2.1 Study Protocol

This retrospective cohort study reviewed 1313 Japanese patients with CLD from Gifu University Hospital. The Institutional Review Board of Gifu University Graduate School of Medicine approved the study protocol (approval number: 2023–233). The study was conducted in accordance with the principles of the Declaration of Helsinki. Informed consent was obtained from the patients using an opt‐out method due to the retrospective nature of the study.

### 2.2 Participants and Follow‐Up

Patients with CLD who were admitted and for whom RTPs were measured between October 2011 and December 2021 were initially included. Exclusion criteria were patients with an active malignancy (including hepatocellular carcinoma), those with an active infection, those who had undergone dialysis, those with a history of transjugular intrahepatic portosystemic shunt insertion, and those who opted out of participation. After discharge, patients were followed up for cirrhosis in the outpatient clinic at least once every 3 months, according to Japanese guidelines [3].

### 2.3 Data Collection

The baseline data, including age, sex, weight, height, etiology of CLD, comorbidities (diabetes mellitus, ascites, hepatic encephalopathy, and sarcopenia), and laboratory data, were extracted from the medical records. Ascites was assessed using medical imaging, and hepatic encephalopathy was evaluated according to the West Haven criteria [13]. The body mass index, Child–Pugh score, model for end‐stage liver disease (MELD) score, and estimated glomerular filtration rate (eGFR) were calculated from the data obtained. Sarcopenia was diagnosed with the criteria proposed by the Japan Society of Hepatology, including reduced skeletal muscle mass index (< 42 cm^2^/m^2^ in males and < 38 cm^2^/m^2^ in females) and handgrip strength (< 28 kg in males and < 18 kg in females) [14]. Survival was determined by the duration from enrollment to death or the last visit as of July 14, 2023.

### 2.4 Measurement of RTPs

RTPs were measured under fasting conditions on the day of admission or the following day. The low RTP group was determined on the basis of previous reports for each sex [15, 16]. Patients with RBP levels below 2.7 mg/dL for males and 1.9 mg/dL for females were included in the low RBP group [15]. The low prealbumin group was defined as those with prealbumin levels below 23 mg/dL and 22 mg/dL for males and females, respectively [16]. The low transferrin group was defined as those with transferrin levels below 190 mg/dL and 200 mg/dL for males and females, respectively [16].

### 2.5 Statistical Analysis

Data were expressed as the median with the interquartile range for continuous variables and as numbers with percentages for categorical variables. The baseline characteristics of the groups were compared using the Mann–Whitney *U* or the chi‐square test. Survival curves were created using the Kaplan–Meier method, and the groups were compared using the log‐rank test. Factors associated with survival were assessed using the Cox proportional hazard regression model, and the results were expressed as hazard ratios (HRs) with 95% confidence intervals (CIs). Factors associated with being in a low RTP group were assessed using the logistic regression model, and the results were presented as odds ratios (ORs) with 95% CIs. The correlation between each RTP and albumin level was evaluated using the Pearson correlation coefficient.

A two‐sided *p* < 0.05 was considered statistically significant. All statistical analyses were performed using the JMP version 17.0.0 software (SAS Institute, North Carolina, America) and R version 4.3.2 software (The R Foundation for Statistical Computing, Vienna, Austria).

## 3 Results

### 3.1 Clinical Characteristics of Enrolled Patients With CLD

**FIGURE 1:**
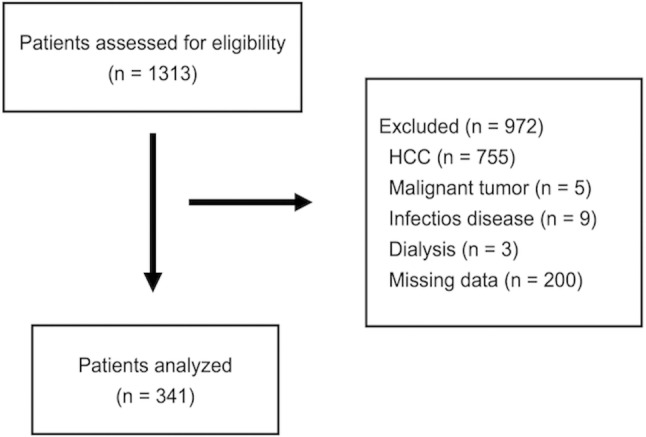
Flow diagram of the study.

Of the 1313 patients screened, 972 were excluded based on the exclusion criteria, and the remaining 341 were included in the analysis (Figure 1). The baseline characteristics of the patients with CLD are shown in Table 1. The median age of the patients was 67 years, and 48% were male. The prevalence of chronic hepatitis, Child–Pugh class A status, Child–Pugh class B status, and Child–Pugh class C status was 25%, 34%, 23%, and 19%, respectively, and the median MELD score was 8 points. The median RBP, prealbumin, and transferrin levels were 1.5 mg/dL, 11 mg/dL, and 227 mg/dL, respectively.

**TABLE 1:** Baseline characteristics of patients with CLD by the RBP level.

	All patients	Low RBP	Normal RBP	*p* *
Characteristics	(*n* = 341)	(*n* = 239)	(*n* = 102)	
Age (years)	67 (55–75)	67 (56–75)	66 (55–74)	0.239
Male, *n* (%)	165 (48)	133 (56)	32 (31)	< 0.001
Body mass index (kg/m^2^)	22.1 (19.8–24.5)	22.0 (19.5–24.8)	22.9 (20.6–24.9)	0.0291
Etiology of CLD, *n* (%)				< 0.001
HCV	122 (36)	82 (34)	40 (39)	
HBV	20 (6)	19 (8)	1 (1)	
ALD	63 (18)	54 (23)	9 (9)	
MASH	39 (11)	18 (8)	21 (21)	
Others	97 (28)	66 (28)	31 (30)	
Diabetes mellitus, *n* (%)	75 (22)	54 (23)	21 (21)	0.682
Ascites, *n* (%)	128 (38)	115 (48)	13 (13)	< 0.001
Hepatic encephalopathy, *n* (%)	31 (9)	29 (12)	2 (2)	0.003
CH/Child–Pugh class A/B/C, *n* (%)	84/116/77/64 (25/34/23/19)	29/80/68/62 (12/33/28/26)	55/36/9/2 (54/35/9/2)	< 0.001
Laboratory test				
MELD score	8 (7–11)	9 (7–12)	6 (6–7)	< 0.001
International normalized ratio	1.06 (0.98–1.21)	1.12 (1.04–1.28)	0.96 (0.92–1.01)	< 0.001
Hemoglobin (g/dL)	11.9 (10.1–13.5)	11.3 (9.6–12.9)	12.9 (11.9–14.3)	< 0.001
Platelet (10^9^/L)	114 (73–173)	94 (67–138)	165 (122–204)	< 0.001
Creatinine (mg/dL)	0.70 (0.57–0.87)	0.70 (0.56–0.89)	0.70 (0.59–0.86)	0.765
eGFR (mL/min/1.73m^2^)	77 (59–91)	79 (61–92)	73 (52–88)	0.064
Albumin (g/dL)	3.3 (2.7–3.9)	3.1 (2.4–3.5)	4.0 (3.6–4.3)	< 0.001
Bilirubin (mg/dL)	1.0 (0.8–1.6)	1.2 (0.9–1.8)	0.8 (0.6–1.0)	< 0.001
Sodium (meq/L)	139 (137–140)	139 (137–140)	139 (138–140)	0.012
Iron (μg/dL)	99 (58–148)	95 (48–152)	103 (71–134)	0.233
Prealbumin (mg/dL)	11 (7–17)	8 (6–12)	18 (15–22)	< 0.001
RBP (mg/dL)	1.5 (1.0–2.3)	1.2 (0.8–1.6)	2.8 (2.2–3.3)	< 0.001
Transferrin (mg/dL)	227 (186–267)	215 (163–261)	252 (216–293)	< 0.001

*Note:* Values are presented as number (percentage) or median (interquartile range).

Abbreviations: ALD, alcohol‐related liver disease; CH, chronic hepatitis; CLD, chronic liver disease; eGFR, estimated glomerular filtration rate; HBV, hepatitis B virus; HCV, hepatitis C virus; MASH, metabolic dysfunction‐associated steatohepatitis; MELD, model for end‐stage liver disease; RBP, retinol‐binding protein.

* Statistical differences between the two groups were analyzed using the chi‐square test or Mann–Whitney *U* test.

Of the patients enrolled in the current study, 70% were diagnosed with low RBP. The low RBP group had a higher proportion of males, a significantly higher complication rate (particularly the prevalence rates of ascites and hepatic encephalopathy), and reduced liver function reserve, as assessed by Child–Pugh class and MELD score. Serum levels of prealbumin and transferrin were also significantly lower in the low RBP group.

### 3.2 Relationship Between RTPs and Mortality in Patients With CLD

**FIGURE 2:**
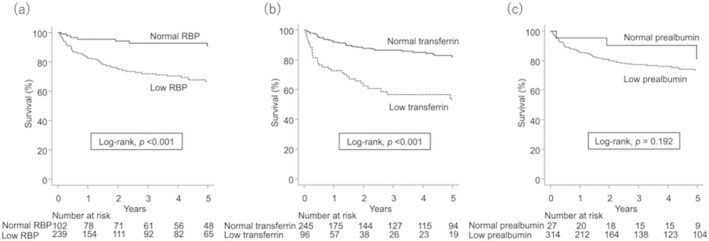
The Kaplan–Meier curve showing the survival rate by (a) RBP levels, (b) transferrin levels, and (c) prealbumin levels. Abbreviation: RBP, retinol‐binding protein.

During the median observation period of 2.3 years, 23% of the patients died. The main cause of death was liver failure (69%), followed by hepatocellular carcinoma (10%). The Kaplan–Meier curve demonstrating the survival rate according to each RTP is shown in Figure 2. The low RBP group had a significantly higher mortality rate than the normal RBP group (1‐year survival rate: 83% vs. 95%; 3‐year survival rate: 72% vs. 93%; 5‐year survival rate: 67% vs. 91%; *p* < 0.001; Figure 2a). Similarly, the low transferrin group had a significantly higher mortality rate than the normal transferrin group (1‐year survival rate: 73% vs. 92%; 3‐year survival rate: 57% vs. 87%; 5‐year survival rate: 54% vs. 82%; *p* < 0.001; Figure 2b). In contrast, no significant difference was found between the low prealbumin group and the normal prealbumin group (1‐year survival rate: 86% vs. 96%; 3‐year survival rate: 77% vs. 90%; 5‐year survival rate: 74% vs. 81%; *p* = 0.192; Figure 2c).

### 3.3 Impact of RTPs on Mortality in Patients With CLD

The results of the multivariate analysis of factors associated with mortality in patients with CLD are shown in Table 2. Among the RTPs, only RBP (HR, 0.62; 95% CI, 0.46–0.81; *p* = 0.002) was significantly associated with mortality in patients with CLD, independent of liver function reserve. Prealbumin (HR, 1.01; 95% CI, 0.98–1.02; *p* = 0.635) and transferrin (HR, 1.00; 95% CI, 1.00–1.00; *p* = 0.709) were not associated with mortality.

**TABLE 2:** Impact of RTPs on mortality in patients with CLD.

Characteristics	HR (95% CI)	*p* *
Age (years)	1.05 (1.03–1.087)	< 0.001
Male	2.76 (1.59–4.77)	< 0.001
Body mass index (kg/m^2^)	0.99 (0.93–1.04)	0.718
Etiology of cirrhosis		
HCV^a^	1.00	
HBV	0.11 (0.01–0.84)	0.033
ALD	0.80 (0.41–1.54)	0.501
MASH	0.92 (0.31–2.71)	0.877
Others	1.44 (0.76–2.71)	0.266
MELD score	1.22 (1.13–1.30)	< 0.001
Sodium (meq/L)	0.91 (0.86–0.98)	0.023
Prealbumin (mg/dL)	1.01 (0.98–1.02)	0.635
RBP (mg/dL)	0.62 (0.46–0.81)	0.002
Transferrin (mg/dL)	1.00 (1.00–1.00)	0.709

Abbreviations: ALD, alcohol‐related liver disease; CI, confidence interval; CLD, chronic liver disease; HBV, hepatitis B virus; HCV, hepatitis C virus; HR, hazard ratio; MASH, metabolic dysfunction‐associated steatohepatitis; MELD, model for end‐stage liver disease; RBP, retinol‐binding protein; RTP, rapid turnover protein.

* Analyses were performed using the Cox proportional hazard model.

^a^ Reference group.

### 3.4 Factors Associated With Low RBP and the Correlation Between RTPs and Albumin in Patients With CLD

**FIGURE 3:**
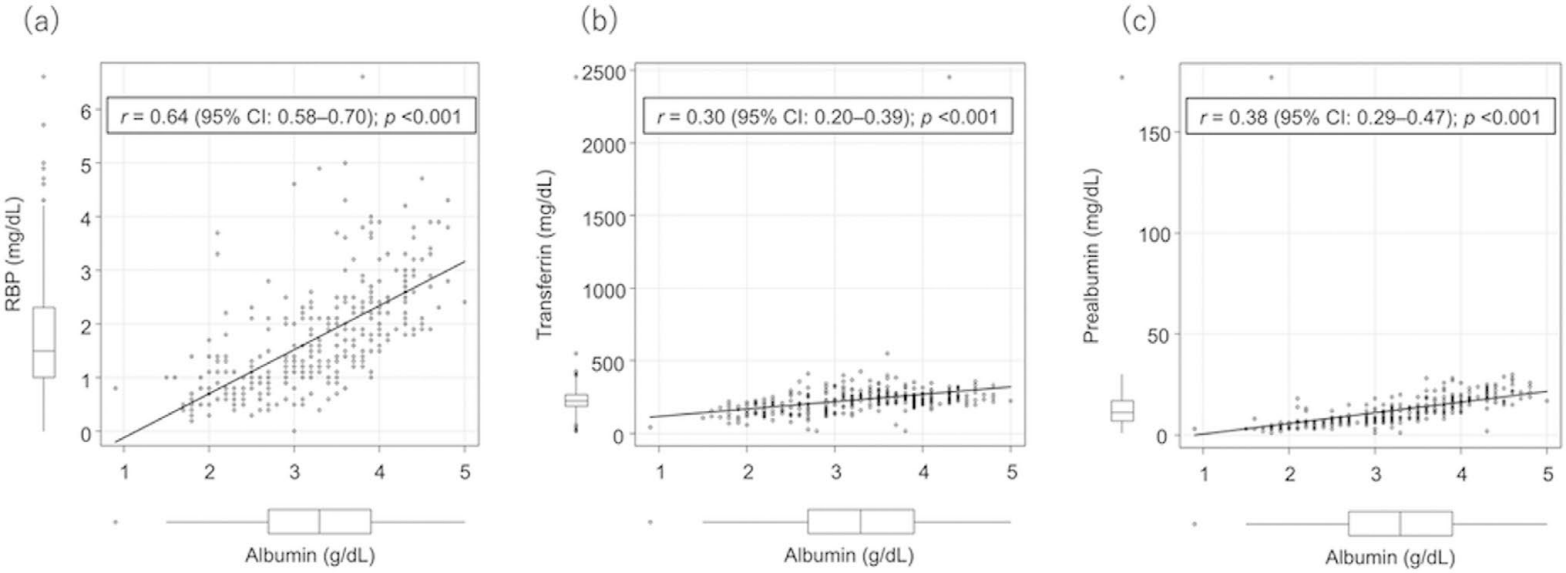
Correlation between albumin and (a) RBP, (b) transferrin, and (c) prealbumin. Abbreviation: RBP, retinol‐binding protein.

Because RBP was associated with mortality in patients with CLD, the factors associated with low RBP were examined. In the multivariate analysis, age (OR, 1.08; 95% CI, 1.03–1.14; *p* = 0.004), male sex (OR, 8.62; 95% CI, 2.56–29.0; *p* < 0.001), BMI (OR, 1.16; 95% CI, 1.00–1.34; *p* = 0.050), serum albumin level (OR, 0.10; 95% CI, 0.04–0.26; *p* < 0.001), and eGFR (OR, 1.08; 95% CI, 1.04–1.11; *p* < 0.001) were significantly associated with low RBP in patients with CLD (Table 3). The correlation between each RTP and albumin was then assessed and found to be strongest with RBP (*r* = 0.64; 95% CI, 0.58–0.70; *p* < 0.001; Figure 3a), whereas its correlation with transferrin (*r* = 0.30; 95% CI, 0.20–0.39; *p* < 0.001; Figure 3b) and prealbumin (*r* = 0.38; 95% CI, 0.29–0.47; *p* < 0.001; Figure 3c) was weak.

**TABLE 3:** Factors associated with low RBP in patients with CLD.

Characteristics	OR (95% CI)	*p* *
Age (years)	1.08 (1.03–1.14)	0.004
Male	8.62 (2.56–29.00)	< 0.001
Body mass index (kg/m^2^)	1.16 (1.00–1.34)	0.050
Etiology of cirrhosis		
HCV^a^	1.00	
HBV	3.96 (0.37–42.30)	0.254
ALD	0.68 (0.17–2.76)	0.586
MASH	2.86 (0.43–18.90)	0.277
Others	2.90 (0.84–9.99)	0.091
MELD score	1.15 (0.91–1.45)	0.251
Albumin (g/dL)	0.10 (0.04–0.26)	< 0.001
Sodium (meq/L)	0.96 (0.78–1.19)	0.725
eGFR (mL/min/1.73m^2^)	1.08 (1.04–1.11)	< 0.001
BCAA (μmol/L)	1.00 (1.00–1.01)	0.663
Sarcopenia^b^	0.46 (0.12–1.73)	0.253

Abbreviations: ALD, alcohol‐related liver disease; BCAA, branched‐chain amino acid; CI, confidence interval; CLD, chronic liver disease; eGFR, estimated glomerular filtration rate; HBV, hepatitis B virus; HCV, hepatitis C virus; MASH, metabolic dysfunction‐associated steatohepatitis; MELD, model for end‐stage liver disease; OR, odds ratio; RBP, retinol‐binding protein.

* Analyses were performed using the Cox proportional hazard model.

^a^ Reference group.

^b^ Data on sarcopenia were missing in 139 cases.

## 4 Discussion

RTPs, which have a shorter half‐life than albumin and reflect protein synthesis and nutritional status, have the potential to be dynamic biomarkers for assessing mortality risk in patients with CLD [6]. However, very few attempts have been made to evaluate RTPs in CLD patients, and their clinical relevance remains poorly understood. The first important finding of this study was that a low level of RBP, a commonly assessed RTP in clinical practice, was associated with mortality in patients with CLD, independent of the function reserve of the liver. The second interesting finding was that age, male sex, BMI, albumin, and eGFR were associated with low RBP, with serum levels of RBP and albumin showing the strongest correlation.

Among RTPs, a previous study suggested that prealbumin levels are associated with postoperative complications in patients undergoing liver transplantation [17]. Prealbumin and transferrin levels have also been reported to be useful in stratifying mortality risk in patients with cirrhosis [18, 19]. However, there is limited evidence of the association between RTPs and mortality in patients with CLD. The present study, which evaluated RBP, prealbumin, and transferrin, provides the first evidence that RBP is the only statistically significant predictor of mortality in patients with CLD. The present results suggest that RBP may be superior to prealbumin and transferrin as a dynamic biomarker for stratifying mortality risk in patients with CLD.

Interestingly, conflicting results have been reported on the association between RBP and mortality in different diseases. Low RBP levels have been reported to be a prognostic indicator in conditions that are strongly associated with malnutrition, such as cancer and chronic obstructive pulmonary disease [20]. RBP is synthesized primarily in the liver and also in other sites such as the kidney, testis, and brain [21]. The primary role of RBP is retinol transport, and retinol itself induces RBP synthesis [21]. Consequently, a reduction in RBP levels in patients with CLD reflects impaired retinol metabolism, which is a robust predictor of liver‐related mortality [12]. In contrast, cumulative evidence has shown that a high RBP level is associated with insulin resistance, type 2 diabetes, and the occurrence of cardiovascular events [22, 23]. Similarly, RBPs have been reported to correlate with serum alanine aminotransferase levels, which reflect inflammation, fibrosis, and hepatocarcinogenesis, in patients with metabolic dysfunction‐associated steatotic liver disease [24, 25]. Obesity is robustly associated with the development and progression of CLD [26]; however, patients with advanced liver disease and obesity have a better prognosis than those without obesity [27]. We presume that a similar paradox may occur in relation to RBP in patients with CLD. Indeed, patients who have undergone posthepatocellular carcinoma resection and have high RBP levels exhibit a higher recurrence rate and worse mortality than those with low RBP levels [28], which can be explained by the relatively preserved liver function reserve and the effects of insulin resistance and hyperinsulinemia in this population [29]. In contrast, the majority of the present study population were patients with cirrhosis who are at a high risk of malnutrition, and a low RBP level was a useful biomarker for predicting short‐term mortality. These results are supported by a previous study, which showed that lower RBP levels are associated with mortality in patients admitted to the intensive care unit with liver disease [30]. Therefore, serum RBP levels should be evaluated to identify those with high mortality risk among patients with CLD who are at risk of malnutrition.

Another finding of this study was that age, male sex, BMI, low serum albumin levels, and high eGFR were associated with low RBP levels in patients with CLD. In general, the normal value of RBP is higher in males than in females [15], and the serum RBP levels are known to be affected by testosterone levels in males but not in females [31]. Because lower testosterone levels due to CLD are prevalent in males but not in females [32], a reduced sex difference in testosterone dynamics may have influenced the results of this study. In this study, male sex was also associated with mortality in patients with CLD. Sex differences should be considered when using RBP as a prognostic factor because sex is also important in assessing nutritional status and sarcopenia in patients with CLD [4]. Regarding renal function, a previous study showed that serum RBP levels were affected by eGFR in older patients with heart failure [33]. Chronic kidney disease is reported to influence hepatic synthesis and secretion of RBP in addition to hydrolysis [34], which can be a reasonable explanation for our study results.

Serum albumin levels are strongly associated with outcomes in patients with CLD [5]. In the present study, serum RBP levels had the strongest correlation with serum albumin levels, suggesting that RBP levels may accurately reflect the severity of liver disease. This finding is also supported by a previous study that showed strong correlations between RBP levels, liver stiffness measurements, and albumin levels in patients with biliary atresia [35]. An accurate assessment of nutritional indicators, including RBP levels, and appropriate nutritional therapy are important to improve the outcome of these patients because increased RBP and albumin levels following nutritional interventions lead to improved survival in patients with CLD [36, 37, 38]. Future studies should evaluate the effectiveness of nutritional interventions based on RBP levels and the clinical relevance of monitoring RBP during treatment.

This study has several limitations. First, the study was conducted in patients with CLD at a single center, which may limit the generalizability of the present results to other regions. Second, the retrospective nature of the study cannot exclude the possibility of bias. Third, there is little evidence of the normal values of RTPs. The number of analyzed cases needs to be increased to establish appropriate cut‐off values for RTPs that define the prognosis of patients with CLD. However, the fact that three RTPs were evaluated in the same cohort, that multivariate analyses were performed with sufficient sample size, and that longitudinal data were used to strengthen the evidence are strengths of the study.

In conclusion, our study revealed that the serum RBP level, a dynamic biomarker of nutritional status, is associated with mortality in patients with CLD, independent of liver function reserve. Further studies are needed to evaluate the efficacy of nutritional interventions based on RBP levels.

## Ethics Statement

The study protocol was reviewed and approved by the Institutional Review Board of the Graduate School of Medicine, Gifu University (approval number: 2023–233).

## Consent

Informed consent was obtained from all the participants using an opt‐out method due to the retrospective nature of the study.

## Conflicts of Interest

The authors declare no conflicts of interest.

## Data Availability

The datasets generated and/or analyzed data during the current study are available from the corresponding author upon reasonable request.
